# Journey of a cystinuric patient with a long-term follow-up from a medical stone clinic: necessity to be SaFER (stone and fragments entirely removed)

**DOI:** 10.1007/s00240-018-1059-5

**Published:** 2018-04-25

**Authors:** Sacha L. Moore, Bhaskar K. Somani, Paul Cook

**Affiliations:** 1 University Hospital Southampton NHS Trust, Southampton, SO16 6YD UK; 2 University of Southampton, Southampton, UK; 3 Medical Stone Clinic, University Hospital Southampton NHS Trust, Southampton, UK

**Keywords:** Cystine, Stone, Urolithiasis, Recurrence, Prevention, Ureteroscopy

## Abstract

There is a lack of studies looking at the longitudinal follow-up of patients with cystine stones. We wanted to assess the journey of cystinuric patients through our specialist metabolic stone clinic to improve the understanding of episodes, interventions and current outcomes in this patient cohort. After ethical approval, all patients who attended our metabolic stone clinic from 1994 to 2014 with at least one cystine stone episode were included in our study. Data were retrospectively analysed for patient demographics, stone episodes or intervention, clinical parameters and patient compliance. Over a period of 21 years, 16 patients with a median age of 15.5 years underwent a mean follow-up of 8.6 years (1–21 years). The mean number of surgical interventions was 3.1 (1–8/patient), but patients who were stone free after their first treatment had lower recurrences (*p* = 0.91) and lower number of interventions during their follow-up (2.7/patient, compared to those who were not stone free at 4/patient). During their follow-up period, patients with < 3 interventions had a significantly better renal function than those with ≥ 3 surgical interventions (*p* = 0.04). Additionally, linear regression analysis showed that eGFR was demonstrated to decline with increasing numbers of stone episodes (*r*^2^ = 0.169). It was also noted that patients who began early medical management remained stone free during follow-up compared to those who had medical management after ≥ 2 stone episodes, of whom all had a recurrent episode. Our long-term longitudinal study of cystine stone formers highlights that patients who are stone free and receive early metabolic stone screening and medical management after their initial presentation have the lowest recurrence rates and tend to preserve their renal function. Hence, prompt referral for metabolic assessment, and the stone and fragments entirely removed (SaFER) principles are key to preventing stone episodes and improving long-term function.

## Introduction

Despite constituting only 1% of adult and 6–10% of paediatric urinary tract calculi [1–3], patients with cystinuria are often the most challenging stone formers to manage [4, 5]. With initial stone presentation often within the first two decades of life and recurrence rates of 60% or greater [6], cystinurics require lifelong treatment and intensive follow-up.

Cystine stones form as the result of defects in one or both of the subunits (rBAT and b^0,+^AT) of the transepithelial sodium-independent antiporter in the renal proximal tubules, causing impaired reuptake of the dibasic amino acids cystine, ornithine, lysine and arginine (COLA) [6–8]. While ornithine, lysine and arginine are quite soluble at a normal physiological urinary pH, cystine is poorly soluble in this pH range with a p*K*a of 8.3 [7, 8]. This low solubility causes supersaturation of cystine in the urine and leads to stone formation.

Dysfunction of this transport mechanism has been attributed to mutations in solute carrier (SLC) genes SLC3A1 and SLC7A9. SLC3A1 at locus 2p16.3 codes for rBAT and mutations in this gene follow an inheritance pattern of autosomal recessive with high penetrance [6, 9, 10]. To date, 177 distinct mutations in SLC3A1 have been identified [11]. Patients with cystinuria as a result of SLC3A1 mutations are referred to as type A cystinurics and constitute approximately 55% of all cystinuric patients in the UK. SLC7A9 at locus 19q13.1 codes for b^0,+^AT and mutations in this gene follow an inheritance pattern on autosomal dominance with incomplete penetrance [10]. To date, 124 distinct mutations in SLC7A9 have been identified [11]. Patients with cystinuria as a result of SLC3A1 mutations are referred to as type A cystinurics and constitute approximately 31% of all cystinurics. A third type, AB, represents those with mutations in both genes.

In recent years, much of the published research around cystinuria has focused on the genetic diversity of this condition [12]. Due to the rarity of this disease, most reports have been with very small sample sizes. Furthermore, there has been a lack of long-term follow-up studies analysing treatment outcomes in this cohort of patients. Therefore, we wanted to assess the journey of cystinuric patients through our specialist stone centre to improve the understanding of current outcomes and long-term follow-up of treatment in this patient group.

## Methods

Following ethical approval (REC reference: 15/LO/1387; IRAS project ID: 187164) for long-term longitudinal follow-up of cystine stone patients, all patients between the ages of 5 and 90 years presenting to our University teaching hospital with at least one cystine stone episode over a period of 21 years between 1994 and 2014 were retrospectively identified (*n* = 16). Electronic patient records were accessed and both clinic letters and investigation results were reviewed.

A database was created with 37 unique categories encompassing patient demographics, stone characteristics, the number of cystine stone episodes before and after referral to our ‘Metabolic stone clinic’, co-morbidities and outcomes of stone treatment with long-term follow-up. A single stone treatment was classed as a single intervention, for example a single percutaneous nephrolithotomy (PCNL), shock wave lithotripsy (SWL) or ureteroscopy (URS). A second spread sheet was created on the database to provide in-depth analysis of each patient consultation with our Consultant Chemical Pathologist in the Metabolic Stones Clinic; this consisted of a further 18 categories including body mass index (BMI), salt and fluid intakes, urinary pH, urinary cystine:creatinine ratio, estimated glomerular filtration rate (eGFR) and patient medications.

Patients were stratified into a number of groups to analyse the full patient journey; this included those who were stone free after their first treatment versus those who required multiple treatments to be stone free, and those who were seen in the specialist Medical Stones Clinic after their first stone episode versus those who were seen after more than one stone episode (due to referral from another centre where specialist medical input had not been available). The stone-free rate (SFR) was ascertained on USS in 13 patients and CT scan in 3 patients.

Analysis of the results was undertaken in both Microsoft Excel and IBM SPSS version 22. Descriptive statistics were calculated on Excel and confirmed on SPSS to ensure accuracy. The Shapiro–Wilk test for normality was used where necessary to check for parametric/non-parametric data. Independent samples *t* test, Mann–Whitney *U* test and Pearson’s Chi-square test were used to check for statistical significance where appropriate.

## Results

**Fig. 1:**
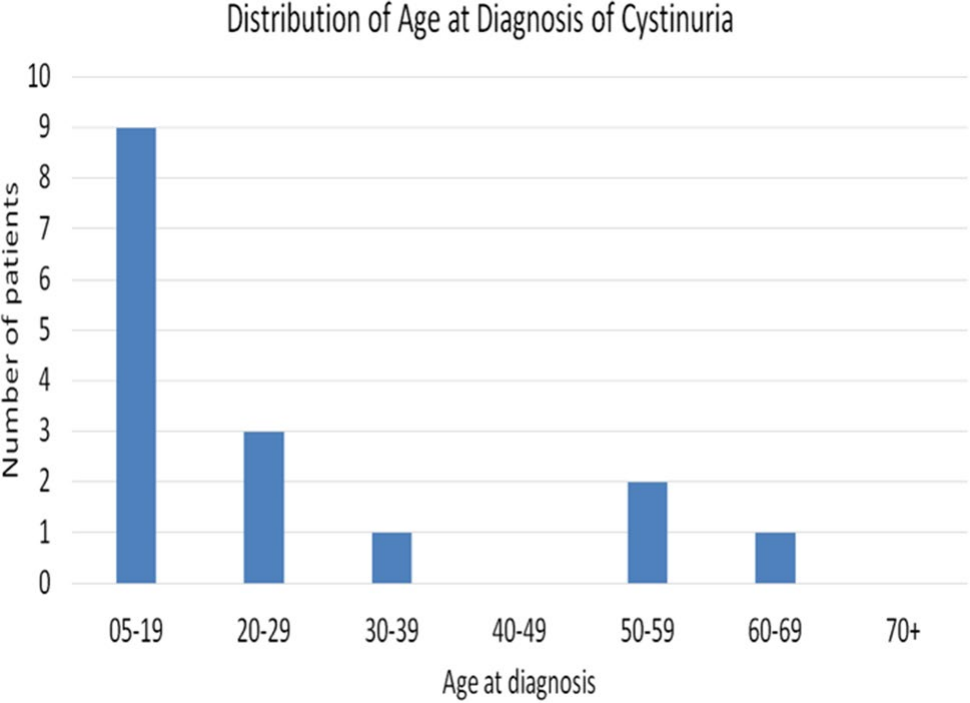
Age at diagnosis of cystine stones

Sixteen patients underwent mean follow-up of 8.6 years (range 1–21 years). Female:male ratio was 1:1. The median age at diagnosis of cystinuria was 15.5 years (range 5–64, IQR 11.50–35.75) (Fig. 1).

### Patient presentation

Information on the location of first presentation was available for 14 individuals; nine patients (64.3%) initially presented to their general practitioner, with just under half of those (4/9) being admitted to the emergency department the same day. Only one patient attended the Emergency Department as their initial presentation. The ratio of patients who lived closer than 5 miles from our centre to patients who lived further than 5 miles away was 1:1.

**Fig. 2:**
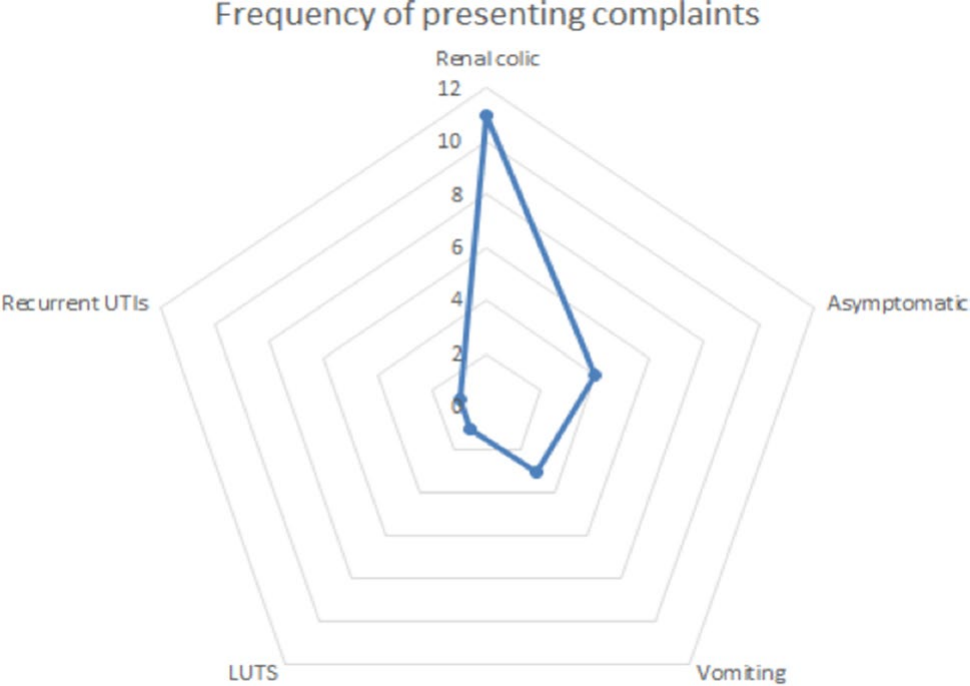
Frequency of presenting complaints

Information on the presenting complaint was available for all patients (Fig. 2); four patients (25.0%) were asymptomatic, while six patients (37.5%) presented with renal colic, abdominal or flank pain as their only symptom. In total, 11 patients (68.8%) presented with some form of pain, 3 (18.8%) complained of vomiting and 1 (6.3%) complained of lower urinary tract symptoms (LUTS). Figure 2 demonstrates the distribution of presenting complaints. There were four staghorn stones, three partial staghorn stones, three lower pole stones, two PUJ stones and four ureteric stones in our study.

### Stone treatment

The surgical treatment in these patients included PCNL in nine patients of which four had PCNL as the solo procedure, URS in six patients of which t had URS as the solo procedure, with most patients having a combination of PCNL, SWL or URS procedure. While SWL was used as a sandwich therapy, it was not used as a solo treatment in any patient. The medical treatment was in form of D-Penicillamine (500 mg twice a day) in four patients, potassium citrate (10 ml three times a day) in five patients and a combination of them in seven patients.

### Outcomes of stone treatment

**Fig. 3:**
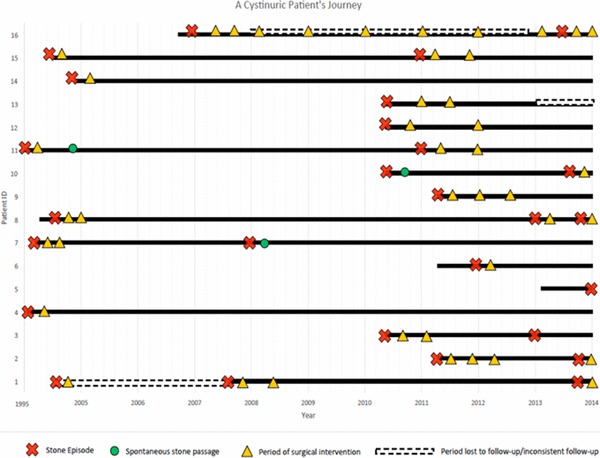
Journey of cystinuria patients

**Fig. 4:**
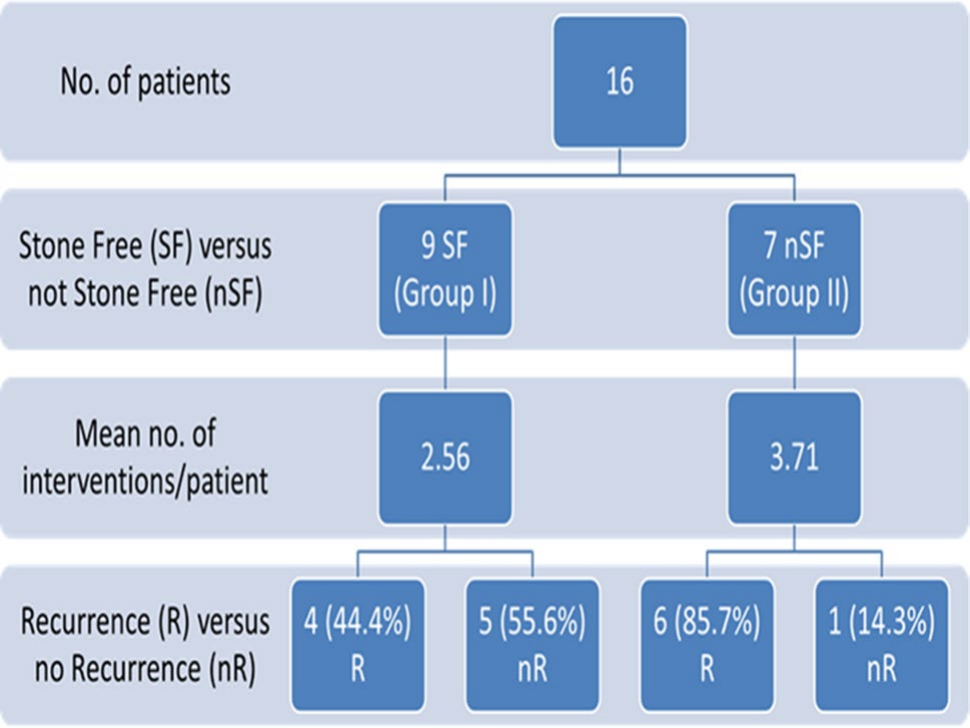
Stone interventions and recurrences based on their initial stone free stats

The mean number of surgical interventions per patient was 3.1 (range 1–8/patient) (Fig. 3). Patients were divided into two groups based on their stone free status after their first treatment (Fig. 4); group I were stone free after one treatment and group II required multiple sessions to be stone free. Stone recurrence was lower in group I compared to group II (*p* = 0.091). The mean number of interventions in group I was also lower (2.75/patient compared to 4.00/patient in group II) (*p* = 0.272). One patient from the stone-free group underwent nephrectomy due to extremely poor renal function and a heavy stone burden at their initial presentation.

Patients were also stratified into two groups according to recurrence status. Ten patients (62.5%) suffered recurrence during the follow-up period. All patients who had a recurrent episode were found to have at least one stone in the same kidney as the initial stone had been. There was no significant difference observed between the number of patients with diagnosed chronic kidney disease and their recurrence status in our cohort.

**Fig. 5:**
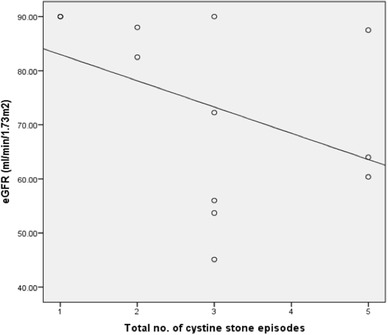
Number of stone episodes and renal function

Renal function data (estimated glomerular filtration rate—eGFR) were available for 12/16 patients due to some patient’s data being documented prior to the introduction of electronic patient records. The mean eGFR was significantly better in patients with < 3 interventions (eGFR − 87.6 mL/min/1.73 m^2^) compared to patients who underwent ≥ 3 surgical interventions (eGFR − 63.1 mL/min/1.73 m^2^) over the follow-up period (*p* = 0.04). Additionally, linear regression analysis showed that eGFR was demonstrated to decline with increasing numbers of stone episodes (*r*^2^ = 0.169) (Fig. 5).

### Outcomes of specialist metabolic evaluation

Data on detailed metabolic evaluation of our cystinuric cohort were available for 12 patients. The mean number of appointments in the Metabolic Stones Clinic per patient per year was 2.02 (SD 0.71). Of those who had BMI data recorded in the clinic (*n* = 10), six (60.0%) were categorised as overweight or obese. Nine patients had both BMI and urinary pH data on record; using the last recorded urinary pH and BMI measurements, those with a BMI > 25 were more likely to have a urinary pH of < 7 (*p* = 0.016). There was no correlation of note between a BMI in the overweight/obese categories and reduced renal function.

Patient compliance was assessed from their urine pH measurements and fluid intake. While the mean urine pH increased from 6.6 (5.2–7.4) to 6.9 (5.4–7.8), the fluid intake was between 3 and 3.5 L in seven patients, 3.5–4 L in five patients, 4.1–4.5 L in three patients and over 4.6 L in one patient. Patients were divided into two groups based on when they started prophylactic treatment to prevent stone recurrence (group III and group IV); group III began appropriate prophylaxis after their first episode, whereas group IV were seen after ≥ 2 stone episodes (Table 1). All patients in group III have remained stone free with no recurrence over a mean follow-up of 9.25 years. All group IV patients have had recurrent stone episodes over a mean follow-up of 8.42 years.

**Table 1:** Shows the difference in stone recurrences between patients who had early versus late metabolic prophylaxis

	Number of patients with recurrence	Number of patients without recurrence	*p* value
Group III (metabolic prophylaxis after first stone treatment)	0	4 (25%)	< 0.05
Group IV (seen after ≥ 2 stone episodes and metabolic prophylaxis after that)	12 (75%)	0	< 0.05

## Discussion

### Meaning of the study

Our study represents a significant landmark in assessing the impact of medical management and long-term follow-up of cystine stone patients. It shows the journey of cystinuric patients with stone episodes and interventions. A female:male ratio of 1:1 highlights the equal spread amongst genders, and the pattern of age at diagnosis is comparable to the literature. The recurrence rate of 62.5% is on par with previously reported values and supports the assertion that cystine stone formers have higher recurrence rates than is average for all stone formers in the same time period [13–15].

Our study also confirms the correlation between raised body mass index and a lower urinary pH that has previously been demonstrated in other stone types [13]. Our data demonstrate evidence of reduced renal function associated with increased surgical intervention in this cohort of patients. It also highlights the potential decline in renal function in patients who have undergone > 3 surgical procedures. Additionally when separated into stone-free and non-stone-free groups, the higher average number of procedures undergone by the non-stone-free group (4.0 vs 2.5 in the stone free group) highlights the need to ensure these patients are entirely stone and fragment free after their first intervention. It is clearly essential to maintain adequate renal function in this cohort of patients for as long as possible and, as such, we suggest the use of the stone and fragments entirely removed (SaFER) concept as a way of prompting clinicians to ensure that patients are stone free.

### Comparison with other studies and the value of medical management

While some studies have questioned the efficacy of medical management in cystine stone formers [14], our data highlight a clear benefit to early specialist medical intervention. In our metabolic stones clinic, patients receive a thorough biochemical work-up, individualised treatment and regular follow-up by a consultant in Chemical Pathology. Our data demonstrate that this intensive specialist approach can help to reduce stone recurrence. Further studies assessing the effect of individualised medical management on quality of life are warranted.

Regarding the surgical management, a similar pattern of recurrence and intervention was seen by another study, with an average of 4.2 interventions/patient [15]. The medical compliance and management in their series of 30 cystinuric patients was shown to be poor with patients experiencing multiple stone episodes requiring multiple interventions. In another Korean study of 14 patients, a multimodal treatment is recommended for a SaFER status [16]. An incidence of recurrence or growth of 3.2/patients was seen over a median follow-up of 5 years in their series despite medical intervention alongside.

In a comparative study of 31 historic patients (1978–1989) undergoing tradition stone treatment (open surgical treatment or nephrectomy) compared to 17 patients treated with minimally invasive treatments (1990 onwards; mainly SWL and PCNL), fewer patients in the modern era underwent nephrectomy or deterioration of renal function with these minimally invasive treatments [17]. An early diagnosis, preventive measures and a comprehensive treatment are recommended to avoid recurrences.

### Strengths and limitations of our study

The strength of our study is the comprehensive medical stone database and the journey of the cystinuric patients, which confirm multiple interventions they need in their treatment. However, with an early diagnosis, medical management and better compliance these patients have reduced episodes of surgical intervention and preserve their renal function in the long term. However, our sample size is small in keeping with the disease prevalence and this has been recognised in other studies [15–17]. As it is a retrospective medical database study and old surgical notes had been archived, full details on intervention type was not available for all patients. Similarly, patients referred from other hospitals had some demographics or information missing.

The effect on Quality of life (QoL) and the cost of treatment were not calculated in this study [18]. A recent study on health-related QoL (HRQoL) demonstrated a lower HRQoL in these patients especially on aspects of social, emotional and disease impact, which is not surprising considering the number of interventions needed in these patients [19]. Although no formal cost was calculated, with the cost of minimally invasive treatments now decreasing [20–22], the overall surgical cost of the journey of these patients is also likely to come down. However, this will only be possible if they maintain their medical compliance and have a rigorous follow-up [23]. Cystinurics tend to have higher number of interventions with a lower QoL compared to other patients [24]. Our study seems to concur with previous recommendation that improved medical management might reduce the number of interventions needed in these patients.

### Areas of future research

Considering all of the studies on cystine stones are retrospective series from individual hospitals, perhaps there is merit in multicentre studies to get meaningful data. Similarly, as each study is from a different unit across the world, there is a lack of standardisation of data collection and reporting process. More must be done to assess patient compliance to medical therapy and ways to improve it [25]. Similarly, a standardised definition of SFR needs to be established for determining the success of their surgical treatment [26].

## Conclusion

Our long-term longitudinal study of cystine stone formers highlights that patients who are stone free and receive early metabolic stone screening and medical management after their initial presentation have the lowest recurrence rates and tend to preserve their renal function. Hence, prompt referral for metabolic assessment, early multidisciplinary input and the stone and fragments entirely removed (SaFER) principles are keys to preventing stone episodes and improving long-term function in this cohort of patients.
